# Supplementary material for: Human Papillomavirus Self-Sampling for Unscreened Women Aged 24 Years During the COVID-19 Pandemic
Source: Healthcare (Basel). 2024 Oct 30;12(21):2160. doi: 10.3390/healthcare12212160 (PMC11545595; doi:10.3390/healthcare12212160)
Supplement: Supplementary file 1 [file healthcare-12-02160-s001.zip › healthcare-3243993-supplementary.pdf]

**Supplementary Table S1**

Female population of Fukui Prefecture based on the 2022 National Census [49]

| | |
|---|---|
| All ages (n) | 329269 |
| 20–24 (n) | 15657 |
| 25-29 (n) | 16346 |
| 30–34 (n) | 18487 |
| 35–39 (n) | 20730 |
| 40–44 (n) | 23764 |
| 45–49 (n) | 27547 |
| 50–54 (n) | 24318 |
| 55–59 (n) | 24515 |
| 60–64 (n) | 24731 |
| 65 (n)- | 133174 |

In Japan, women aged 20 years and older are advised to undergo cytological screening for cervical cancer every two years. Half of the women in this age group are eligible for cervical cancer screening.
